# Impact of a Ration Negative in Dietary Cation–Anion Difference and Varying Calcium Supply Fed before Calving on Colostrum Quality of the Dams and Health Status and Growth Performance of the Calves

**DOI:** 10.3390/ani10091465

**Published:** 2020-08-20

**Authors:** Abbas Rajaeerad, Gholam Reza Ghorbani, Mohammad Khorvash, Ali Sadeghi-Sefidmazgi, Amir Hossein Mahdavi, Saman Rashidi, Mirja Rosmarie Wilkens, Martin Hünerberg

**Affiliations:** 1 Department of Animal Sciences, College of Agriculture, Isfahan University of Technology, Isfahan 84156-83111, Iran; a.rajaeerad@ag.iut.ac.ir (A.R.); ghorbani@cc.iut.ac.ir (G.R.G.); khorvash@cc.iut.ac.ir (M.K.); sadeghism@cc.iut.ac.ir (A.S.-S.); mahdavi@cc.iut.ac.ir (A.H.M.); saman.rashidi@alumni.iut.ac.ir (S.R.); 2 Department of Animal Sciences, Animal Nutrition Physiology, University of Goettingen, Kellnerweg 6, 37077 Goettingen, Germany; 3 Department of Animal Sciences, Ruminat Nutrition Unit, University of Goettingen, Kellnerweg 6, 37077 Goettingen, Germany; martin.huenerberg@uni-goettingen.de

**Keywords:** prepartum dairy cows, dietary cation–anion difference, Ca restriction, colostrum, neonate calves, body weight development

## Abstract

**Simple Summary:**

We fed diets with low and high levels of calcium or a negative dietary cation–anion difference (DCAD) to dairy cows before calving and compared their colostrum quality and the health and growth performance of their calves. There were no differences in birth weight and colostrum quantity and composition among groups. However, calves born to cows fed a diet with a negative DCAD had lower body weight and higher prevalence for diarrhea before weaning. The level of Ca in the diet of the dams before calving had no impact on the body weight, feed intake, and health of the calves.

**Abstract:**

This study investigated the effect of diets negative in dietary cation–anion difference (DCAD) or restricted in Ca fed prepartum to dairy cows for three weeks on colostrum yield and composition, and the health and growth performance of their calves. Thirty-six pregnant non-lactating Holstein-Friesian cows were randomly assigned to three isoenergetic diets: (1) low Ca: 0.24% Ca, DCAD: +86 mEq/kg; (2) high Ca: 1.23% Ca, DCAD: +95 mEq/kg; and (3) low DCAD: 1.28% Ca, DCAD: −115 mEq/kg (all dry matter (DM) basis). While colostrum quality was not affected, low Ca supply prepartum tended to increase the colostrum yield compared to high Ca (low Ca = 8.81 vs. high Ca = 5.39 kg). However, calves from cows fed low DCAD showed higher serum concentrations of K, lower body weight (BW), starter feed intake and average daily weight gain before weaning compared to low Ca and high Ca calves (53.12 vs. 57.68 and 57.32 kg) but BW was similar postweaning (d 70). In addition, calves from dams fed low DCAD were more likely to develop diarrhea and had increased number of days with abnormal fecal scores. Consequently, calves from low DCAD dams had to be treated more frequently.

## 1. Introduction

Supplementing dairy cows with anionic salts during the last three weeks before calving (i.e., close-up period) improves Ca homeostasis, decreases the risk of retained placenta, metritis, and enhances the overall health status during the early postpartum phase [1,2]. The adverse effect of a negative dietary cation–anion difference (DCAD) on prepartum feed intake is also well documented [1,2,3]. Compromised dry matter intake (DMI) during late gestation is frequently associated with decreased colostrum production and reduced fetal growth [4,5,6]. However, findings whether a small or moderate decrease in DMI (5–15%) in close-up cows fed low-DCAD diets could affect colostrum production or fetal growth are limited and inconsistent [2,3].

For example, reducing the DCAD from −70 to −180 mEq/kg diet dry matter (DM) decreased colostrum yield by 1.4 kg but had no effect on the birth weight of calves [7]; whereas increasing the duration during which the low DCAD diets were fed from 21 to 42 d prepartum, decreased the birth weight by 2.8 kg [8]. In contrast, feeding a low DCAD diet containing −157.9 mEq/kg DM did not affect prepartum DMI, fetal growth, and colostrum yield compared to the control (+188.5 mEq/kg DM [9]).

Calves from hypocalcemic cows have a greater risk of developing diarrhea and respiratory infections compared to calves born from non-hypocalcemic dams [10,11]. Based on these findings, it could be speculated that improving Ca homeostasis in dairy cows around calving by lowering their DCAD during the close-up phase might have a positive impact on the health of calves.

Feeding diets formulated to have a negative DCAD induces a compensated metabolic acidosis, which can lead to decreased blood and urine pH and altered the blood gas concentrations [3,12]. Even relatively small changes of the maternal acid-base balance may cause fetal distress in humans, which can affect the function of the fetal central nervous and cardiovascular system [13]. The placental nutrient transfer system of cows is highly vascularized, particularly during the last trimester [14]. Therefore, it is possible that a maternal metabolic acidosis could affect the development of the calf in utero and compromise its neonatal health and growth performance. Respiratory acidosis by prolonged or intermittent asphyxia in utero has been associated with reduced efficiency of colostral Ig absorption and increased mortality [15,16,17]. On the other hand, a manipulation of dietary mineral supplementation during the prepartum period has been shown to increase blood immunoglobulin A (IgA) and IgM in the cows, their colostrum and their newborn calves [18].

Several studies reported that the absorption of IgG in neonatal calves was negatively affected by prepartum exposure of their dams to low-DCAD diets [19,20,21,22]. In contrast, feeding two levels of negative DCAD (−70 or −180 mEq/kg DM) and for 21 or 42 d prepartum did not lead to changes in the efficiency of IgG transfer between treatments [8]. However, feeding cows with diets containing −70 or −180 mEq/kg DM for 42 d induced a transient metabolic acidosis in their calves and decreased their BW at birth and day 62 compared to feeding for only 21 days prepartum [8].

Recently, our group found that restricting the Ca level in the diet of prepartum cows to 0.24%, together with a reduction of K (1.14%; both DM basis), is an effective means to reduce the occurrence of periparturient hypocalcemia [23]. However, feeding a low DCAD diet (−115 mEq/kg DM) decreased milk protein content during the first 21 d of lactation, which may have a negative impact on the colostrum quality.

To our knowledge, there is no research examining the effect of a prepartum Ca restriction of dairy cows on colostrum yield and composition, and the physiology of their calves. Therefore, the objectives of this experiment were to determine if feeding low DCAD diets during the prepartum period affects the colostrum quality and quantity of the dams or blood metabolites, health and performance of the calves, and to determine whether a Ca restriction of close-up cows has any advantageous effects on colostrum quality and health and growth performance of their calves in comparison low DCAD close-up diets.

## 2. Materials and Methods

The study was conducted from March 2017 to September 2017 at the Lavark Research Station of Isfahan University of Technology (IUT; Isfahan, Iran). Before the start of the study, the experimental protocol was reviewed and approved by the IUT institutional animal care committee. Cows and calves were cared for in accordance with the guidelines of the Iranian Council of Animal Care.

### 2.1. Experimental Design and Treatment Diets

Thirty-six non-lactating multiparous Holstein-Friesian cows were moved into individual sand-bedded pens 21 days before their expected calving date. The cows were assigned to 3 dietary treatments based on their previous 305-d milk yield, BW, body condition score (BCS), and parity. Treatments were: (1) low Ca: 0.24% Ca, 1.14% K, and DCAD: +86 mEq/kg DM; (2) high Ca: 1.23% Ca, 1.17% K, and DCAD: +95 mEq/kg DM; and (3) low DCAD = high Ca diet supplemented with 5.51% SoyChlor (Mehre-Bistun Corporation, Tehran, Iran) 1.28% Ca, 1.16% K, and DCAD: −115 mEq/kg DM (all diet DM basis). Diets were formulated to meet the nutrient requirements of prepartum dairy cows using the Cornell Net Carbohydrate and Protein System (CNCPS Version 5.1, Cornell University, Ithaca, NY, USA) and to be isonitrogenous and isoenergetic (Table 1). Cows were fed once per day for an ad libitum intake. Further details concerning the management of the cows are described by Rajaeerad et al. [24].

### 2.2. Calving and Calf Management

Calving ease was scored using a 4-point scale (1 = no assistance needed; 2 = light assistance by one person without the use of mechanical traction; 3 = mechanical extraction of the calf with an obstetric calf-puller; and 4 = severe dystocia, surgery or fetotomy needed) according to [27]. Calves were removed from their dams immediately after birth, weighed, and the calves’ navels were dipped prophylactically in 2%-iodine solution. Calves were transferred to individual pens (2.9 × 1.1 m; length × width) located in an open-sided barn equipped with large ceiling fans. The calf pens were bedded with straw.

On their day of birth (d 0), all calves consumed 3.5 L of colostrum from their dams in two feedings (2 L within 1 h after birth and 1.5 L 8 h later) by nipple bottle. From day 1 to 49, calves received 6 L of pasteurized whole milk containing 3.25% ± 0.12% fat, 2.98% ± 0.06% protein, 4.88% ± 0.05% lactose, and 11.77% ± 0.15% total solids twice daily at 09:00 h and 19:00 h. Starting on day 50 the quantity of milk was reduced to 3 L of milk/d and the calves were only fed once (at 09:00 h). Calves were weaned on day 56 but remained in individual pens until the end of the experiment (70 d). The calves had access to a dry TMR (i.e., calf starter) containing 90% concentrate and 10% alfalfa hay (Table 1). Starter and fresh water were offered for ad libitum intake from d 1 until the end of experiment.

### 2.3. Colostrum Collection and Analyses

Dams were milked within 30 min after calving, the colostrum was weighted and two colostrum samples were taken. One sample (ca. 250 mL) was used to measure the specific gravity with a colostrometer (Funke-Gerber Labortechnik GmbH, Berlin, Germany). The specific gravity, determined at 22 °C, was used to estimate the Ig content of the colostrum [28]. A Brix refractometer (Model LH-Y12, Atago Co., Ltd., Tokyo, Japan) was used to determine the concentration of IgG [29]. A second sample (30 mL) was frozen at −20 °C to measure the colostrum composition. After thawing, colostrum samples were diluted (1:5, vol:vol) with phosphate buffer saline (pH 7.3) and homogenized by ultrasound mixing (5 min). Samples were analyzed for fat, protein, lactose, and solids-not-fat (SNF) by an infrared analyzer (MilkoScan BN, Foss Electric, Hillerød, Denmark).

### 2.4. Blood Analyses 

Blood samples from each calf were collected by jugular vein puncture using a 10 mL serum Vacutainer^®^ (Becton Dickinson, Franklin Lakes, NJ, USA), approximately 20 min before colostrum feeding on d 0 and 1, 7, 35, and 70 at 3 h after the morning feeding. The blood samples were stored at room temperature to allow clotting. Samples were subsequently centrifuged at 3000× *g* for 15 min. The serum was decanted into three aliquots and stored at −20 °C until analysis. Serum samples were analyzed for Ca, Mg, and P by the cresolphthalein-complexone method, xildyl-blue method, and UV-test, respectively, using a diagnostic kit (Pars Azmoon Co., Tehran, Iran). The kit was used according to the manufacturer’s instructions. Samples were analyzed using an auto analyzer (Alcyon 300, Abbott Laboratories, Abbott Park, IL, USA).

The serum glucose concentration was measured by an enzymatic assay (GOD-PAD, Pars Azmoon Co., Tehran, Iran). Blood urea-N (BUN), total protein, albumin, alanine aminotransferase (ALT), and aspartate aminotransferase (AST) were measured using commercial kits (Pars Azmoon Co., Tehran, Iran) according to the manufacturer’s instructions. All analyses were performed in duplicates using an autoanalyzer (Abbott Alycon 300, Abbott Park, IL, USA,). Before any samples were processed, the analyzer was calibrated with control sera containing known concentrations of N and P (TrueLab N^®^ and TrueLabP^®^, Pars Azmoon Co., Tehran, Iran) and a standard calibration solution (TrueCal U^®^, Pars Azmoon Co., Tehran, Iran). Beta-hydroxybutyrate (BHB) concentrations were determined by an enzymatic colorimetric method using a commercial kit (Randox Laboratories Ltd., Ardmore, UK) and the same autoanalyzer. Globulin concentrations were calculated by subtracting the albumin from the total protein content.

### 2.5. Feed Intake, Body Weight, and Analyses 

Starter feed offered and refused was recorded weekly for each calf and used to calculate average daily feed intake. Starter feed and refusals were sampled once per week during the entire experiment. Samples of the starter feed (pooled by week) and refusals (pooled monthly by calf) were frozen at −20 °C until analyzed. Milk intake was recorded for each feeding. The BW of the calves was recorded at birth and once per week during the preweaning (d 0–55) and postweaning phase (d 56–70). Preweaning, postweaning, and overall means for ADG and feed efficiency (FE; kg BW gain/kg total DM intake (milk DM + starter feed DM)) were calculated.

Prior to analyses, feed samples were ground through a 1-mm screen using a Wiley mill (Arthur Thomas Co., Philadelphia, PA). Dry matter was determined by oven-drying at 65 °C until constant weight was reached. For CP analysis (N × 6.25), samples were analyzed by Kjeldahl titration (Kjeltec 1030 Auto Analyzer, Tecator, Höganäs, Sweden; [30]); method 988.05. Ether extract (EE) and ash contents were analyzed according to [30]; method 920.39 and 924.05, respectively. Neutral detergent fiber was analyzed according to [31] using heat stable α amylase (100 μL/0.5 g of sample) and sodium sulfite. All chemical analyses were performed in duplicates.

### 2.6. Health Monitoring, Fecal Scoring, and Medical Treatment

Calf health was monitored daily by a veterinarian who was unaware of the treatment assignment. The consistency of the feces was scored daily on a scale of 0–3, (0 = normal consistency, 1 = semi formed or pasty, 2 = loose, and 3 = watery, sifts through bedding) using the Wisconsin–Madison calf health scoring chart [32]. To describe the duration of diarrhea, the number of days with an abnormal fecal score (AFS ≥ 2) was recorded for each calf.

Calves with diarrhea (fecal score ≥ 2) received a water-based oral electrolyte solution (6 L/calf per day in 4 equal meals for three consecutive days) and sulfadimidine sodium (220 mg/kg BW for 3 consecutive days; 6 mL/10 kg BW; Tolide Darouhai Dami Co., Tehran, Iran). Non-responding calves were treated for three more days with oxytetracycline (Oxyject L. A.; 1 mL/10 kg BW on the first 2 d; Rooyan Darou Co., Tehran, Iran) and Pantrisul (40 mg Trimethoprim and 200 mg Sulfamethoxazole per mL, 1 mL/10 kg BW on the first two days; Pantex Holland B.V., Hapert, The Netherlands). To treat pneumonia, calves were administered oxytetracycline (Tenaline 20% L. A.; 1 mL/10 kg BW on day 1 and 3; Ceva Sante Animale, Libourne, France), florfenicol (F-nex 300; 1.5 mL/10 kg BW for three consecutive days; Razak Laboratories Co., Karaj, Iran), and flunixin meglumine (Flunixin 5%; 1 mL/10 kg BW for three consecutive days; Razak Laboratories Co., Karaj, Iran). Drug usage (type of medication, frequency, and duration of the treatment) was recorded throughout the experiment.

### 2.7. Statistical Analysis

All statistical analyses were conducted using SAS (version 9.4, SAS Institute Inc., Cary, NC, USA). Data were subjected to an ANOVA using the MIXED procedure (Proc Mixed). Feed intake, ADG, feed conversion efficiency, BW, and blood variables were sampled at several time points and therefore sampling time (day) was considered as repeated measure for the preweaning (day 0–55) and postweaning (day 56–70) phase as well as the complete duration of the experiment (day 0–70). The statistical model included fixed effects of treatment, sex, period, and interaction among treatments and period and calf as a random effect. Birth weight and colostrum data were analyzed with the same model described above but without repeated measures and the effect of sampling time. The effect of dietary treatment on the categorical responses (fecal scores and drug usage) was analyzed by PROC GLIMMIX with repeated measures. The best-fitting covariance structure (autoregressive structure type 1) was selected based on the lowest Akaike information criterion corrected for sample size [33]. Results are presented as least squares means (LSM). 

Differences among treatment LSM were determined using a Tukey test. Treatment differences were declared significant at *p* < 0.05. Tendencies are discussed at 0.05 ≤ *p* < 0.10. 

## 3. Results

### 3.1. Calving Ease, Colostrum Yield, and Composition

Prepartum Ca restriction tended to result in a higher yield of colostrum (low Ca = 8.8 kg, Table 2) compared to cows in the high Ca group (5.3 kg; *p* = 0.06), while feeding low DACD had no effect on colostrum production. Treatments did not affect the percentage of fat, protein, lactose, and SNF but yields of fat, lactose, and SNF (kg) increased in response to low Ca (*p* < 0.01). Cows offered high Ca produced less true protein in comparison to low Ca (*p* < 0.01).

The Brix value and specific density of the colostrum were not affected by the dietary treatment (Table 3). Calving ease was similar among treatments

### 3.2. Mineral and Intermediary Metabolism

The Ca level or DCAD of the prepartum diet did not affect the serum concentrations of Ca, P, and Mg of the calves during the preweaning and postweaning phase or the complete length of the experiment (Table 4). There was no interaction between treatment and time of these parameters. Serum concentrations of Ca and P were elevated at birth and day 1 (*p* < 0.01) but decreased subsequently until day 70.

Before weaning (day 0–55), calves born to low DCAD dams had greater serum K concentrations (4.44 mg/dL) compared to those born to high Ca (4.16 mg/dL) and low Ca (4.13 mg/dL) dams (*p* < 0.001) but there were no differences in serum K concentrations among treatments after weaning.

There was no effect of dietary treatment or the interaction between treatment and age for total serum protein, albumin, and globulin concentrations during preweaning, postweaning, or the entire length of the experiments (Table 5). In addition, there was no effect of dietary treatment on BUN.

Indices related to energy metabolism, such as glucose and BHB, did not differ among treatments before weaning (Table 4). After weaning and over the entire length of the experiment, BHB concentrations increased markedly (*p* < 0.001).

### 3.3. Feed Intake and Growth Performance

Calves born to cows receiving the low DCAD diet consumed less starter and total feed (starter and milk combined) during the first week (*p* < 0.01). Treatment had no effect on total DMI and starter feed intake during the preweaning or postweaning and the overall experiment.

**Figure 1:**
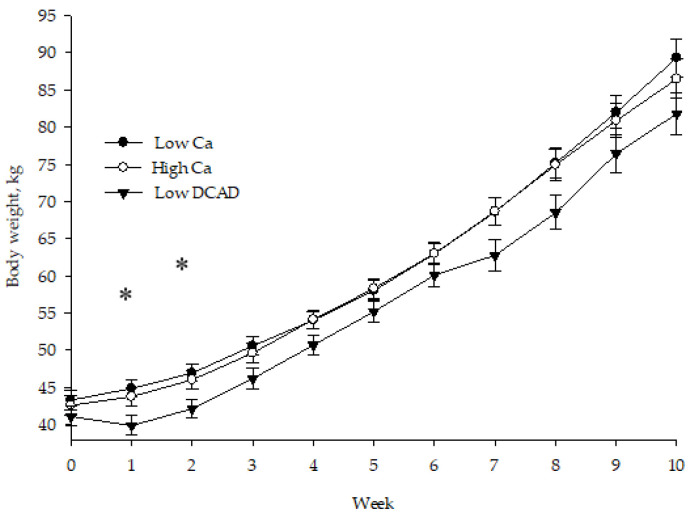
Body weight development of neonate calves from Holstein cows fed low Ca, high Ca, or a negative dietary cation–anion difference (low DCAD) for 21 d before calving. * Significant differences between groups are indicated by asterisks (*p* < 0.05).

The birth weight of the calves (Table 6) was similar among treatments (43.32, 42.63, and 41.06 kg for calves born to low Ca, high Ca, and low DCAD); while calves born to low DCAD diet had lower BW in wk 1 in comparison to calves born to low Ca and high Ca cows (Figure 1; *p* = 0.01; 39.30 vs. 45.04 and 43.67 kg). In addition, they had lower BW at wk 2 (*p* = 0.02) compared to low Ca (42.55 vs. 47.09 kg). Due to this, there was an interaction (*p* = 0.01) between treatment and age of the calves for BW during preweaning (*p* = 0.04) and the entire length of the experiment (*p* = 0.02). Body weight at weaning (day 56) tended (*p* = 0.08) to be lower for calves born to cows that received low DCAD compared to calves born to low Ca and high Ca cows (69.13 vs. 75.48 and 74.91 kg, respectively). Post weaning BW was similar at day 70.

**Figure 2:**
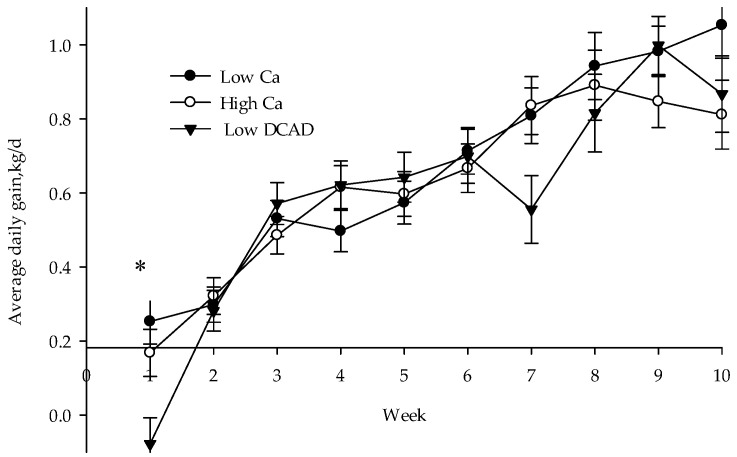
Body weight development of neonate calves from Holstein cows fed low Ca, high Ca, or a negative dietary cation–anion difference (low DCAD) for 21 d before calving. * Significant differences between groups are indicated by asterisks (*p* < 0.05).

Average daily gain was not affected by treatment during the pre- and postweaning phase and the whole experiment but there was an interaction (*p* = 0.02) between treatment and age of the calves for ADG. Calves born to a low DCAD diet had lower ADG in week 1 in comparison to calves born to low Ca and high Ca cows (Figure 2; *p* = 0.01; −0.07 vs. 0.22, 0.17 kg). A tendency for lower ADG (*p* = 0.07) was observed at week 7 of study in calves born to a low DCAD cow as well.

No differences in feed efficiency were detected between treatments and interactions between treatment and time during the preweaning, postweaning, and overall periods (*p* > 0.11).

### 3.4. Fecal Scores and Medication Usage

Calves from dams fed low DCAD prior to calving had higher mean fecal scores before weaning and over the length of the entire experiment (*p* < 0.05; Table 7). Similarly, calves from low DCAD dams suffered from diarrhea for longer, as indicated by the increased number of days with AFS (≥2; *p* < 0.01). The number of days calves needed to be medicated also increased as a result of feeding a low DCAD (*p* = 0.04). The prepartum Ca restriction of cows (high Ca vs. low Ca) had no impact on the fecal scores or the number of days calves needed to be medicated.

## 4. Discussion

Feeding and management practices of prepartum cows affect not only the health and production performance of the dams [34] but also the quantity and composition of their colostrum, as well as the health and performance of their offspring in utero and after birth [6,14]. However, studies examining the effects of dietary changes during the close-up phase on the health and growth performance of calves in a systematic fashion are scarce. Although numerous studies reported improved Ca hemostasis, production performance, and less metabolic disorders in dairy cows in response to feeding low DCAD diets during the close-up phase, only few examined the impact of acidogenic diets fed to cows during late gestation on their colostrum and the health growth performance of their calves.

Results of this study indicate that feeding negative DCAD diets to close-up cows has no impact on the colostrum yield and composition compared to high Ca or low Ca. In agreement with our result, other studies reported that supplementation of anionic salts prepartum had no adverse effect on colostrum yield [9,35,36]. For example, reducing the DCAD of prepartum diets from approximately +130 to −130 mEq/kg DM did not change colostrum quantity or its composition [36].

We did not quantify IgG, as most important colostrum quality parameter, but we are confident that the Brix value is sensitive enough to be used as proxy for colostrum quality. Brix values are highly correlated with colostrum IgG concentrations determined by a radial immunodiffusion assay [29] and Brix readings of ≥21% are routinely used as a threshold to screen for colostrum with high enough IgG concentration to transfer passive immunity (≥50 g IgG/L [21]). The finding that the Brix value and colostrum density were not affected by prepartum diets in our experiment is in agreement with several other studies, who reported that lowering the DCAD of prepartum cows did not change IgG concentration of their colostrum [7,35,36].

The reason for the tendency for higher colostrum yields in response to low Ca at first milking is somewhat unclear. Higher DMI of cows receiving low Ca (15.7 kg/d) compared to high Ca (14.4/d kg) and low DCAD (13.7 kg/d; [23]) might have increased nutrient availability and stimulated colostrum production but colostrogenesis is complex and not only driven by nutrient supply but foremost by endocrine factors and local mechanisms within the mammary gland [37]. Diehl et al. [35] reported that prepartum Ca supply ranging from 1.8 to 1.3% diet DM did not change the colostrum yield but affected IgG concentration and Brix value. Overall, there is only limited data concerning the impact of the dietary Ca level on colostrum yield and composition. This is surprising, since it is common practice to restrict the Ca intake of dairy cows before calving to reduce their risk for hypocalcemia.

We hypothesized that the reduced prepartum DMI, frequently observed in response to low DCAD diets, might negatively affect fetal growth and subsequently the calves’ birth weight. Although DMI of cows offered low DCAD decreased by 2.0 kg/d compared to low Ca [23], differences in birth weight among calves from dams fed low DCAD (41.1 kg) and low Ca (43.3 kg) were only numerical. This is in line with Weich et al. [9], who found no difference in birth weight of calves from cows fed a diet containing −160 mEq/kg of DM (41.1 kg) compared to calves from cows fed +120 mEq/kg DM (44.6 kg). Similarly, Diehl et al. [35] reported that feeding a diet containing −200 mEq/kg DM for 28 d prepartum had no effect on the birth weight of calves compared to the control (−30 mEq/kg DM).

Reduced DMI during the late stage of gestation does not always have negative consequences for the birth weight of calves. For example, restricting beef cows to 70% of their net energy requirements for the last 40 days prior to calving did not affect the weight of their calves [38]. Collazos et al. [8] reported that extending the duration of feeding acidogenic diets (−70 and −180 mEq/kg DM) from 21 to 42 d prepartum reduced the average birth weight of calves from 42.9 to 40.0 kg; which is similar to the numerical difference in birth weight between calves from low Ca and low DCAD cows (−2.2 kg) in this study. This suggests that reduced nutrient transfer or incorporation into fetal tissues in response to feeding DCAD can occur but the extent of the reduction might depend on how far the DCAD is lowered and how long the cows are fed low DCAD diets.

The reduction in starter feed intake of calves from dams fed low DCAD, particularly during week 1 and 2, was one of the reasons for their lower BW before weaning. Based on a model developed to predict the growth of Holstein calves, starter intake is one of the most important factors affecting ADG and BW around weaning [39].

There are only a limited number of studies examining the effect of reduced Ca supply or low DCAD in close-up cows on the feed intake and BW development of neonatal calves. In addition, there are no studies that compare the impact of the two relatively common prepartum feeding strategies on health and growth performance of calves. In contrast to our results, Collazos et al. [8] reported that a reduction in DCAD from −70 to −180 mEq/kg DM did not affect the BW and ADG of calves at the age of 21, 42, and 62 days, whereas extending the duration of feeding from day 21 to 42 prepartum reduced the BW of the calves at birth and day 62. It is important to note that [8] did not collect data from birth until 21 days of age, which is different compared to our study. Similar to our results, Collazos et al. [8] reported that the compromised BW of calves from dams fed low DCAD before weaning was compensated for after weaning.

Collazos et al. [8] also measured blood pH, pCO_2_, and HCO_3_^−^ of neonatal calves and reported that calves born to cows fed low DCAD diets (−70 and −180 mEq/kg DM) were in a state of metabolic acidosis for three days after their birth. Extending the duration of feeding low DCAD diets from 21 to 42 days before calving exerted a more severe metabolic acidosis on the calves. However, calves from dams fed a control, non-acidogenic diet were not included in that study. Another difference is that Collazos et al. [8] fed the newborn calves with pooled colostrum, while we used colostrum of each individual dam for the first two feedings of her respective calf. This might have been a factor of influence as colostrum contains many bioactive substances that were not measured in this study but may impact the growth, morphology, and functional maturation of the gastrointestinal (**GI**) tract, as well as the intermediary metabolism and endocrine system of neonatal calves [40,41,42]. Mann et al. [43] found that changes of the energy level of prepartum diets can lead to changes in colostrum composition beyond IgG concentration, such as insulin concentration and fatty acid composition and that these compounds have an impact on the morphology of the intestinal epithelium of neonatal calves. The altered acid base balance promotes changes in vascular smooth muscle tone, which can influence circulation and blood pressure control [44]. Changes in blood flow to the mammary gland during colostrogenesis can affect the composition of colostrum [45]. In sows, lowering the dietary DCAD from 226 to 170 meq/kg during gestation increased the concentrations of serum and colostrum Ca on the first day of lactation [46]. Therefore, it is justified to speculate that the some of the findings in the present study could have been caused by differences in colostrum composition beyond the measured constituents.

Charbonneau et al. [3] reported that lowering the DCAD by about 300 mEq/kg DM induced a metabolic acidosis in prepartum dairy cows (lowered blood HCO_3_^−^ concentration and urinary pH) that was not fully compensated for. An induced short-term (30 min) acidosis (blood pH = 7.06) in humans did not alter fetal vein pH but led to an efflux of total CO_2_ from the placenta into maternal circulation [47]. Therefore, it is possible that a metabolic acidosis of the dam induced by a negative DCAD could induce an acidosis in utero and affects postnatal BW development as the nutrient transfer system of the uterus is highly vascularized, particularly during the last trimester.

Postnatal respiratory or metabolic acidosis in calves can reduce the absorption efficiency of immunoglobulins in calves, even though they receive adequate amounts of colostrum shortly after birth [21,48]. Hypercapnia in apparently healthy newborn calves was also associated with reduced absorption of IgG from colostrum [49]. In contrast, metabolic acidosis in calves born to cows fed low DCAD diets prepartum did not change the efficiency of IgG absorption [8]. Similarly, calves from dams receiving anionic salts before calving −100 mEq/kg DM) had similar serum IgG concentrations (15.1 vs. 14.4 g/L) and apparent efficiency of absorption compared to calves born from control dams [38].

Unfortunately, transfer of IgG to the calves was not measured in the current study but the serum concentration of total protein on days 0, 1, 7, 35, and 70 for calves from dams fed low DCAD was similar in low Ca and high Ca calves. Passive transfer of immunity to calves can be assessed directly by measuring serum IgG or estimated based on the total protein concentration in the serum [50,51]. A failure of passive transfer can be assumed if total serum protein concentrations are <5.2 g/dL [52]. A total serum protein concentration of calves in this study was 6.42 ± 0.42 g/dL at day 1 and was similar among treatments. Therefore, it can be assumed that the lower preweaning BW and reduced growth performance of calves from dams fed DACD was not caused by insufficient IgG transfer and a lack of passive immunity.

Calves in this study were generally healthy and we had no mortalities. Only one calf (high Ca group) suffered from a respiratory infection. However, calves born to cows fed low DCAD had higher fecal scores, increased number of days with AFS (≥2), and they required medication more frequently, especially during week 1 and 2. Similar to low Ca (3.87) and high Ca calves (4.25) in this study, Bateman et al. [39] reported that an AFS of 4.7 days was common for neonate Holstein calves. In contrast, calves born to dams fed low DCAD in this study experienced 7.93 days with AFS. Unsurprisingly, growth performance of calves is negatively affected by an increase in the number of days with diarrhea [39]. When calves are sick, energy is needed for the immune response and less energy is partitioned towards growth [53]. Furthermore, inflammation of the GI tract and increase in digesta passage in response to diarrhea commonly lead to a reduction of nutrient absorption [54]. Similarly, to reduced growth performance, occurrence of diarrhea in dairy calves can have negative impact on first lactation performance and be associated with reduced milk yield [55].

In contrast to our results, feeding a negative DCAD (−70 and −180 mEq/kg DM) to close-up cows in a previous study did not change the percentage of young calves that had to be treated for diarrhea [8]. However, the apparent discrepancy between Collazos et al. [8] and our study should to be interpreted carefully. We scored the feces of the calves daily between day 1 and 70, whereas Collazos et al. [8] only recorded fecal scores on day 21 and 42. Typically, calves suffer from diarrhea during the first 28 days of life, while the most common period is the first 21 days [56].

Although Ca, Mg, and P concentrations in the serum of the calves were not affected by the dietary treatment of the dams, serum K concentration on day 1 and 7 increased in calves born to low DCAD dams. This could be indicative of compensation of metabolic acidosis as well as an inflammation of the GI tract and loss of buffer through feces. Hyperkalemia is a common electrolyte disturbance in diarrheic calves [57,58,59] and blood pH, base excess, and HCO_3_^−^ were lower, whereas serum K and Na were elevated in diarrheic calves compared to clinical healthy calves [60]. Trefz et al. [61] reported that serum potassium concentrations >5.8 mmol/L were more closely associated with dehydration and diarrhea than with decreases in base excess or venous blood pH.

Unfortunately, we did not measure the acid–base balance or blood pH of the calves in our study. Therefore, conclusions in this regard are largely speculative. However, the mean urine pH of cows fed low DCAD (pH 5.6) was lower than commonly observed (pH 6–7; [3,62]) and also lower compared to cows fed low Ca (pH 7.81) and high Ca (pH 8.14). This would indicate a challenge of the acid–base balance of low DCAD dams, which might have also affected the fetuses. The average urine pH of cows fed low DCAD in the current experiment was similar to the urine pH reported by Collazos et al. [8] in response to feeding −180 mEq/kg diet DM. Calves born to cows fed the ration containing −180 mEq/kg DM experienced metabolic acidosis for three days after birth [8]. This supports the assumption that calves from dams fed low DCAD in the current study may have been in a state of metabolic acidosis, which might have affected their health, increased the likelihood of developing diarrhea, and impacted their growth performance until weaning. Further research would be required to evaluate the long-term effect of this on the performance of heifers during first lactation, as for every 100 g decrease in BW before weaning; first lactation milk yield can be expected to decrease by 155 kg [63].

## 5. Conclusions

Feeding low DCAD or Ca restricted during the close-up phase did not change the colostrum quantity or composition, while cows fed low Ca tended to produce more colostrum compared to dams fed high Ca before calving. Feeding low DCAD to prepartum cows increased the likelihood of diarrhea and serum K concentrations of their calves before weaning. Calves born to dams fed low DCAD during close-up also had lower BW and ADG during the first two weeks and tended to have lower BW before weaning compared calves from cows fed high Ca and low Ca. The delay in the growth development observed in calves born to dams fed low DCAD was compensated after weaning (d 55–70).

## Figures and Tables

**Table 1:** Ingredient and chemical composition of the prepartum diets and calf starter.

Item	Prepartum Diet ^1^			Calf Starter
	Low Ca	High Ca	Low DCAD	
Ingredient, % of DM				
Corn silage	34.96	34.49	34.58	0
Wheat straw, chopped	16.84	15.36	16.33	0
Alfalfa hay	0	0	0	10.0
Barley grain, ground	8.35	5.92	5.49	7.3
Corn, ground	17.22	19.2	18.52	56.2
Soybean meal	7.56	10.0	0	23.2
Canola meal	12.6	10.20	14.68	0
Palm fat	0.46	0.46	0.95	0
Mineral supplement ^2^	0.76	0.76	0.76	0.4
Vitamin supplement ^3^	0.76	0.76	0.76	0.4
SoyChlor ^4^	0	0	5.51	0
Salt	0.21	0.21	0.19	0.5
Magnesium oxide	0.32	0.35	0.3	0
Calcium carbonate	0	2.30	1.84	1.2
Sodium bicarbonate	0	0	0	0.8
Chemical composition ^5^				
DM, %	45.2	44.7	46.4	90.9
CP, %	14.1	14.1	14.1	20.2
NDF, %	42.2	40.5	39.7	16.1
NFC ^6^, %	34.5	34.1	32.6	51.3
EE, %	3.1	3.1	3.8	3.2
Ca, %	0.24	1.23	1.21	0.82
P, %	0.39	0.38	0.37	0.39
Mg, %	0.44	0.42	0.49	0.22
K, %	1.14	1.17	1.21	1.13
S, %	0.32	0.30	0.40	0.20
Na, %	0.11	0.11	0.10	0.49
Cl, %	0.24	0.24	0.76	0.36
DCAD ^7^, mEq/kg	86	95	−115	231
NEL ^8^, Mcal/kg	1.49	1.48	1.48	
ME ^9^, Mcal/kg	2.31	2.29	2.29	2.76

^1^ Low Ca diet (0.24% Ca, +86 mEq/kg); high Ca diet (1.23% Ca, +95 mEq/kg), low dietary cation–anion difference (DCAD; high Ca diet supplemented with SoyChlor; 1.28% Ca, −115 mEq/kg; all dry matter (DM) basis). ^2^ The cow mineral contained per kilogram DM: 16,000 mg Zn, 10,000 mg Mn, 4000 mg Cu, 800 mg Fe, 150 mg I, 120 mg Co, and 80 mg Se. The calf mineral contained per kilogram DM: 240,000 mg Ca, 67,500 mg Mg, 20,000 mg Zn, 4290 mg Cu, 200 mg I, and 100 mg Co. ^3^ The vitamin supplement for the cows contained per kilogram DM: 1,000,000 IU vitamin A, 360,000 IU vitamin D3, and 15,000 IU vitamin E. The vitamin supplement included in the calf starter contained (IU/kg DM) vitamin A, 1,300,000; vitamin D, 360,000; and vitamin E, 12,000. ^4^ Contained 46% crude protein, 12.1% Cl, 1.2% Ca, 0.4% Mg, 0.32% S, and 1.15% K (all DM basis; Mehre Bisotun Corporation, Tehran, Iran). ^5^ Determined on samples pooled by week. All values except DM are expressed on a DM basis (*n* = 8). ^6^ Non-fiber carbohydrate 100—((NDF—neutral detergent insoluble CP) + CP + ash + fat). ^7^ Dietary cation–anion difference ((Na^+^ + K^+^) − (Cl^−^ + S^–2^)). ^8^ Based on tabular values (Cornell Net Carbohydrate and Protein System; [25]). ^9^ Calculated based on the chemical analysis [26].

**Table 2:** Colostrum yield and composition of Holstein cows fed diets fed low or high Ca or a negative dietary cation–anion difference (DCAD) for 21 d before calving (*n* = 12).

Item	Treatment ^1^			SEM	*p*-Value
	Low Ca	High Ca	Low DCAD		
First milking (colostrum)					
Yield, kg	8.81	5.39	6.75	1.40	0.06
Fat, %	4.81	5.07	4.94	0.10	0.17
True protein, %	14.4	14.6	14.7	0.14	0.33
Lactose, %	3.7	3.6	3.6	0.06	0.45
Solids-not-fat, %	17.8	18.1	18.0	0.15	0.31
Fat, kg	0.40 ^a^	0.27 ^b^	0.32 ^b^	0.02	<001
True protein, kg	1.22 ^a^	0.80 ^b^	0.99 ^ab^	0.08	<001
Lactose, kg	0.31 ^a^	0.20 ^b^	0.24 ^b^	0.02	<001
Solids-not-fat, kg	1.55 ^a^	0.98 ^b^	1.20 ^b^	0.10	<001

^1^ Low Ca diet (0.24% Ca, +86 mEq/kg); high Ca diet (1.23% Ca, +95 mEq/kg), low DCAD (high Ca diet supplemented with SoyChlor; 1.28% Ca, −115 mEq/kg; all DM basis). ^a,b^ Different superscripts indicate significant differences between the respective groups.

**Table 3:** Colostrum quality, calving ease of Holstein cows fed low or high Ca or a negative dietary cation–anion difference (DCAD) for 21 days before calving and the rectal temperature of their calves at birth (*n* = 12).

Item	Treatment ^1^			SEM	*p*-Value
	Low Ca	High Ca	Low DCAD		
Brix value colostrum, %	25.83	27.26	26.34	1.5	0.82
Colostrum density, g/cm^3^	1.057	1.059	1.061	2.1	0.27
Calving ease ^2^	1.25	1.33	1.33	0.20	0.80
Rectal temperature at birth, °C	38.26	38.14	38.47	0.12	0.52

^1^ Low Ca diet (0.24% Ca, +86 mEq/kg); high Ca diet (1.23% Ca, +95 mEq/kg), low DCAD (high Ca diet supplemented with SoyChlor; 1.28% Ca, −115 mEq/kg; all DM basis). ^2^ Recorded using a 4-point scale (1 = no assistance needed; 2 = light assistance by one person without the use of mechanical traction; 3 = mechanical extraction of the calf with an obstetric calf-puller; and 4 = severe dystocia, surgery or fetotomy needed).

**Table 4:** Blood chemistry parameters of calves from Holstein dams fed low or high Ca or a negative dietary cation–anion difference (DCAD) for 21 days before calving (*n* = 12).

Item ^1^	Treatment ^2^			SEM	*p*-Value		
	Low Ca	High Ca	Low DCAD		Trt.	Day	Trt × Day
Glucose, mg/dL							
Preweaning (d 0–55)	97.86	93.74	105.63	6.2	0.37	0.11	0.69
Postweaning (d 70)	94.54	96.93	99.87	2.6	0.78		
Overall (d 0–70)	97.06	94.54	100.83	4.4	0.59	0.05	0.27
Beta-hydroxybutyrate, mM							
Preweaning (d 0–55)	0.18	0.17	0.19	0.01	0.14	<0.01	0.15
Postweaning (d 70)	0.27	0.30	0.34	0.02	0.32		
Overall (d 0–70)	0.19	0.20	0.20	0.01	0.35	<0.01	0.24
AST ^3^, U/L							
Preweaning (d 0–55)	26.82	27.85	25.33	1.7	0.58	0.82	0.43
Postweaning (d 70)	38.20	41.54	37.13	5.0	0.82		
Overall (d 0–70)	29.67	31.25	28.38	1.56	0.46	<0.01	0.94
ALT ^4^, U/L							
Preweaning (d 0–55)	9.48	9.50	8.73	0.63	061	0.08	0.55
Postweaning (d 70)	13.24	15.43	12.67	1.6	0.50		
Overall (d 0–70)	10.44	10.93	9.75	0.58	0.34	<0.01	0.69
Ca, mM							
Preweaning (d 0–55)	3.03	3.12	2.98	0.09	0.33	0.01	0.39
Postweaning (d 70)	2.94	2.82	2.70	0.09	0.40		
Overall (d 0–70)	3.02	3.04	2.91	0.07	0.44	<0.01	0.22
P, mM							
Preweaning (d 0–55)	2.42	2.55	2.63	0.07	0.24	<0.01	0.09
Postweaning (d 70)	2.64	2.57	2.68	0.11	0.70		
Overall (d 0–70)	2.53	2.56	2.65	0.06	0.52	<0.01	0.08
Mg, mM							
Preweaning (d 0–55)	0.79	0.78	0.74	0.02	0.21	0.25	0.06
Postweaning (d 70)	0.76	0.72	0.71	0.04	0.69		
Overall (d 0–70)	0.78	0.77	0.74	0.02	0.15	0.19	0.20
K, mM							
Preweaning (d 0–55)	4.13 ^b^	4.16 ^b^	4.44 ^a^	0.06	0.001	<0.01	<0.01
Postweaning (d 70)	3.86	3.97	3.81	0.09	0.51		
Overall (d 0–70)	4.06 ^b^	4.09 ^b^	4.28 ^a^	0.08	<0.01	<0.01	<0.01

^1^ Blood samples were collected before the first colostrum feeding and on d 1, 7, 35, and 70 before feeding. ^2^ Calves born to cows fed low Ca diet (0.24% Ca, +86 mEq/kg), high Ca diet (1.23% Ca, +95 mEq/kg), and low DCAD (high Ca diet supplemented with SoyChlor; 1.28% Ca, −115 mEq/kg; all DM basis) for 21 d before calving. ^3^ Aspartate aminotransferase. ^4^ Alanine aminotransferase. ^a,b^ Different superscripts indicate significant differences between the respective groups.

**Table 5:** Protein metabolism parameters of calves (*n* = 12) from Holstein dams fed low or high Ca or a negative dietary cation–anion difference (DCAD) for 21 days before calving (*n* = 12).

Item ^1^	Treatment ^2^			SEM	*p*-Value		
	Low Ca	High Ca	Low DCAD		Trt	Day	Trt × Day
Total protein, mg/dL							
Preweaning (d 0–55)	6.89	7.20	7.23	0.21	0.36	<0.01	0.21
Postweaning (d 56–70)	7.33	7.02	7.4	0.28	0.59		
Overall (d 0–70)	6.93	7.13	725	0.19	0.48	<0.01	0.40
Albumin, mg/dL							
Preweaning (d 0–55)	3.05	3.27	3.23	0.09	0.17	<0.01	0.14
Postweaning (d 56–70)	3.63	3.76	3.86	0.10	0.24		
Overall (d 0–70)	3.20	3.38	3.40	0.07	0.12	<0.01	0.12
Globulin, mg/dL							
Preweaning (d 0–55)	3.84	3.96	3.90	0.20	0.19	<0.01	0.32
Postweaning (d 56–70)	3.70	3.26	3.50	0.25	0.50		
Overall (d 0–70)	3.72	3.74	3.86	0.17	0.83	<0.01	0.41
Albumin:globulin							
Preweaning (d 0–55)	0.86	0.90	0.88	0.05	0.91	<0.01	0.24
Postweaning (d 55–70)	1.03	1.18	1.15	0.08	0.36		
Overall (d 0–70)	0.90	0.97	0.95	0.04	0.63	<0.01	0.36
Urea nitrogen, mg/dL							
Preweaning (d 0–55)	27.9	32.3	31.2	2.13	0.16	0.21	0.60
Postweaning (d 70)	27.8	29.3	31.3	1.47	0.22		
Overall (d 0–70)	27.8	33.1	31.0	1.73	0.10	0.35	0.48

^1^ Blood samples were collected before the first colostrum feeding and on d 1, 7, 35, and 70 before feeding. ^2^ Calves born to cows fed low Ca diet (0.24% Ca, +86 mEq/kg); high Ca diet (1.23% Ca, +95 mEq/kg), and low DCAD (high Ca diet supplemented with SoyChlor; 1.28% Ca, −115 mEq/kg; all DM basis) for 21 d before calving.

**Table 6:** Body weight, average daily gain, starter feed intake, and feed efficiency of calves from Holstein dams fed low or high Ca or a negative dietary cation–anion difference (DCAD) for 21 days before calving (*n* = 12).

Item	Treatment ^1^			SEM	*p*-Value		
	Low Ca	High Ca	Low DCAD		Trt	Day	Trt × Day
Starter intake, kg DM/d							
Preweaning (d 0–55)	0.39	0.40	0.38	0.04	0.88	<0.01	0.05
Postweaning (d 56–70)	1.98	1.63	1.58	0.19	0.20	<0.01	0.88
Overall (d 0–70)	0.71	0.64	0.62	0.05	0.51	<0.01	0.39
Total DMI, kg/d							
Preweaning (d 0–55)	1.03	1.04	1.01	0.04	0.91	<0.01	0.02
Postweaning (d 56–70)	1.98	1.63	1.58	0.19	0.20	<0.01	0.88
Overall (d 0–70)	1.23	1.16	1.14	0.06	0.55	<0.01	0.53
Body weight ^2^, kg							
Initial (d 0)	43.32	42.63	41.06	1.31	0.48		
At weaning (d 55)	75.48	74.91	69.13	2.0	0.08		
End of study (d 70)	89.58	86.45	82.37	3.71	0.21		
Preweaning (d 0–55)	57.68 ^a^	57.32 ^a^	53.12 ^b^	1.32	0.05	<0.01	0.04
Postweaning (d 56–70)	85.70	83.65	79.94	2.54	0.32	<0.01	0.15
Overall (d 0–70)	63.24	62.56	58.42	1.57	0.08	<0.01	0.02
ADG, kg/d							
Preweaning (d 0–55)	0.58	0.55	0.38	0.08	0.29	<0.01	0.46
Postweaning (d 55–70)	1.01	0.83	0.94	0.07	0.22	0.53	0.24
Overall (d 0–70)	0.66	0.62	0.59	0.03	0.30	<0.01	0.02
Feed efficiency ^3^							
Preweaning (d 0–55)	0.56	0.46	0.37	0.08	0.32	<0.01	0.29
Postweaning (d 56–70)	0.53	0.60	0.65	0.06	0.56	<0.01	0.14
Overall (d 0–70)	0.55	0.55	0.52	0.02	0.63	<0.01	0.11

^1^ Calves born to cows fed low Ca diet (0.24% Ca, +86 mEq/kg); high Ca diet (1.23% Ca, +95 mEq/kg), and low DCAD (high Ca diet supplemented with SoyChlor (1.28% Ca, −115 mEq/kg; all DM basis) for 21 d before calving. ^2^ Body weight was measured weekly throughout the experiment. ^3^ Feed efficiency was calculated by dividing average daily gain by average total daily DM intake. ^a,b^ Different superscripts indicate significant differences between the respective groups.

**Table 7:** Fecal scores and number of medication days of calves from Holstein dams fed low or high Ca or a negative dietary cation–anion difference (DCAD) for 21 days before calving (*n* = 12).

Item	Treatment ^1^			SEM	*p*-Value		
	Low Ca	High Ca	Low DCAD		Trt	Day	Trt × Day
Fecal score ^2^							
Preweaning (d 0–55)	1.02 ^b^	1.04 ^b^	1.10 ^a^	0.01	0.01	<0.01	0.22
Postweaning (d 56–70)	1.03	1.04	1.04	0.01	0.87	0.02	0.18
Overall (d 0–70)	1.02 ^b^	1.04 ^b^	1.08 ^a^	0.01	0.02	<0.01	0.15
Abnormal fecal score ^3^, d	3.87 ^b^	4.25 ^b^	7.93 ^a^	0.65	0.001		
Calves diagnosed at least once for diarrhea, *n*	7/12	8/12	11/12				
Days medicated	1.16 ^c^	1.50 ^bc^	2.98 ^ab^	0.48	0.04		

^1^ Calves born to cows fed low Ca diet (0.24% Ca, +86 mEq/kghigh Ca diet (1.23% Ca, +95 mEq/kg), and low DCAD (high Ca diet supplemented with SoyChlor; 1.28% Ca, −115 mEq/kg; all DM basis) for 21 d before calving. ^2^ Feces consistency was scored daily on a scale of 0–3, (0 = normal consistency, 1 = semiformed or pasty, 2 = loose, and 3 = watery, sifts through bedding) using the Wisconsin–Madison’s Calf Health Scoring chart [32]. ^3^ Days with score ≥ 2. ^a,b,c^ Different superscripts indicate significant differences between the respective groups.
